# The Diversity and Evolution of *Wolbachia* Ankyrin Repeat Domain Genes

**DOI:** 10.1371/journal.pone.0055390

**Published:** 2013-02-04

**Authors:** Stefanos Siozios, Panagiotis Ioannidis, Lisa Klasson, Siv G. E. Andersson, Henk R. Braig, Kostas Bourtzis

**Affiliations:** 1 Department of Environmental and Natural Resources Management, University of Western Greece, Agrinio, Greece; 2 Department of Molecular Evolution, Uppsala University, Uppsala, Sweden; 3 School of Biological Sciences Bangor University, Bangor Gwynedd, United Kingdom; 4 Biomedical Sciences Research Center Al. Fleming, Vari, Greece; 5 Insect Pest Control Laboratory, Joint FAO/IAEA Division of Nuclear Techniques in Food and Agriculture, Vienna, Austria; University of Poitiers, France

## Abstract

Ankyrin repeat domain-encoding genes are common in the eukaryotic and viral domains of life, but they are rare in bacteria, the exception being a few obligate or facultative intracellular Proteobacteria species. Despite having a reduced genome, the arthropod strains of the alphaproteobacterium *Wolbachia* contain an unusually high number of ankyrin repeat domain-encoding genes ranging from 23 in *w*Mel to 60 in *w*Pip strain. This group of genes has attracted considerable attention for their astonishing large number as well as for the fact that ankyrin proteins are known to participate in protein-protein interactions, suggesting that they play a critical role in the molecular mechanism that determines host-*Wolbachia* symbiotic interactions. We present a comparative evolutionary analysis of the *w*Mel-related ankyrin repeat domain-encoding genes present in different *Drosophila*-*Wolbachia* associations. Our results show that the ankyrin repeat domain-encoding genes change in size by expansion and contraction mediated by short directly repeated sequences. We provide examples of intra-genic recombination events and show that these genes are likely to be horizontally transferred between strains with the aid of bacteriophages. These results confirm previous findings that the *Wolbachia* genomes are evolutionary mosaics and illustrate the potential that these bacteria have to generate diversity in proteins potentially involved in the symbiotic interactions.

## Introduction

*Wolbachia* is a group of intracellular and maternally transmitted alphaproteobacteria, comprising strains with diverse symbiotic relationships with numerous arthropod as well as filarial nematode species [1], [2]. These bacteria are quite widespread in insects and crustaceans [3]. Recent screens suggest that >40% of all terrestrial arthropod species are infected, rendering *Wolbachia* perhaps the most abundant symbiotic microorganism of the biosphere [4].

The successful spread of *Wolbachia* into insect host populations has been attributed to their unique ability to act as manipulators of host reproduction in order to ensure their own transmission. *Wolbachia* infections have been associated with the induction of feminization, thelytokous parthenogenesis, male killing and, most commonly, cytoplasmic incompatibility (CI) [1], [2], [5]. During the last decade, several studies have shown that *Wolbachia* infections can affect, in addition to reproduction, several additional aspects of host biology, physiology, ecology and evolution [1], [2], [5], [6]. The molecular mechanisms that allow *Wolbachia* to establish symbiotic associations and to induce its extended phenotypes are yet to be unraveled.

Advances in genomics provided significant new information about *Wolbachia* biology. There are currently four complete genome sequences of *Wolbachia,* while several others are either available as permanent drafts or in progress [2], [7]–[13]. A common feature of all insect *Wolbachia* genomes is the high percentage of repetitive elements such as insertion sequences, group II introns, duplicated segments of prophages and multi-gene families such as the ankyrin repeat genes [14]–[16].

Ankyrin repeat domains (ANK hereafter) consist of a tandem motif of about 33 amino acids and act as scaffolds that mediate protein-protein interactions [17], [18]. Proteins with ANK domains are commonly found in eukaryotes and viruses, but they can also, although rarely, be found in the Bacteria and Archaea [19]. ANK proteins are known to be involved a multitude of functions, such as cell-cycle regulation, transcriptional regulation, cytoskeleton interactions, signal transduction, development, intracellular trafficking, sex differentiation, and they can also act as toxins [17], [18], [20]. Recent findings suggest that ANK proteins represent a new family of bacterial type IV effectors that play a major role in host-pathogen interactions and the evolution of infections [21], [22]. Indeed, it was shown that ANK proteins of certain pathogenic intracellular bacteria are secreted into the host cytoplasm and interact with host factors [22]–[25]. The intracellular pathogen *Anaplasma phagocytophilum*, for example, secrets AnkA through a type IV secretion system (T4SS). The protein interacts with specific regions of the host chromatin, resulting in a modulation of host gene transcription [23], [24], [26]–[28].

The potential role of ANK proteins in *Wolbachia* symbiosis and the manipulation of host reproduction has been investigated in *Drosophila* and mosquito species, but no direct correlation of the *Wolbachia* ankyrin gene repertoire with any bacterial phenotype has so far been established [10], [29]–[34]. It is worth noting, however, that (a) the presence of certain ANK gene variants has been associated with crossing types in *Culex quinquefasciatus* [32] and (b) some ANK genes are under host sex-specific regulation [31]. A recent large-scale proteomic analysis of the excretory-secretory products of the *Wolbachia* infected filarial parasite *Brugia malayi* identified the presence of two *Wolbachia*-encoded ankyrin proteins [35]. These are probably the first *Wolbachia* ANK proteins shown to be secreted. *Wolbachia* also carries a functional T4SS [9], [10], [13], [36], strengthening the hypothesis that the ANK genes play a role in functional and evolutionary processes of host-*Wolbachia* symbiosis.

Genomic analysis showed that the ANK genes account for up to 4% of the total number of genes in the insect *Wolbachia* strains *w*Mel, *w*Ri and *w*Pip [9], [10], [13]. Additionally, comparative analyses between the ANK genes found in these genomes showed that they often evolve very rapidly, through for example gene duplication and contraction/expansion of repeated sequences within ANK domains [9], [10]. Taken together, the above led to the suggestion that the ANK proteins may play a pivotal role in the molecular interaction between host and symbiont [9], [10], [13], [30].

To learn more about the genetic diversity and the molecular mechanisms that underlie the evolution of the *Wolbachia* ANK gene family, we investigated the distribution of *w*Mel ANK genes in several different *Drosophila*-*Wolbachia* associations, analyzed their genetic diversity by sequencing the identified orthologs and reconstructed their phylogenetic relationship. Our results indicate that both homologous and illegitimate recombination along with genomic flux provided by prophages and transposable elements are key factors in generating polymorphism and shaping the ANK gene pool in the *Wolbachia* strains studied.

## Materials and Methods

### Ethics Statement

N/A.

### Fly Lines and *Wolbachia* Strains

The *Drosophila* lines and *Wolbachia* strains used in this study are listed in [37]–[45] Table 1. Flies were grown at 24°C ±1 on cornflour/sugar/yeast medium. *Wolbachia* strains were chosen according to their modification/rescue phenotype (mod^+^/resc^+^, mod^−/^resc^+^, mod^−/^resc^−^) and their embryonic localization pattern (posterior, anterior or uniform) as described by Veneti et al. [44]. The *Wolbachia* infection status of the *Drosophila* lines used was confirmed based on *wsp* and MLST typing as well as on CI properties [46]–[48].

**Table 1:** *Drosophila* lines and *Wolbachia* strains.

*Wolbachia* supergroup	Host species	*Wolbachia* strain	Embryonic distribution	CI phenotype	Reference
A	*D. melanogaster*	*w*Mel	posterior	mod^+^ resc^+^	[38], [40], [44]
	*D. melanogaster*	*w*MelPop	posterior	mod^+^ resc^+^	[43], [44]
	*D. simulans*	*w*Au	posterior	mod^−^ resc^−^	[42], [44]
	*D. teissieri*	*w*Tei	posterior	mod^−^ resc^+^	[44], [45]
	*D. yakuba*	*w*Yak	posterior	mod^−^ resc^+^	[44], [45]
	*D. santomea*	*w*San	posterior	mod^−^ resc^+^	[44], [45]
	*D. simulans*	*w*Ri	uniform	mod^+^ resc^+^	[41], [44]
	*D. simulans*	*w*Ha	uniform	mod^+^ resc^+^	[41], Veneti pers. comm.
B	*D. simulans*	*w*No	anterior	mod^+^ resc^+^	[41], [44]
	*D. mauritiana*	*w*Mau	anterior	mod^−^ resc^+^	[39], [44]
	*D. simulans*	*w*Ma	anterior	mod^−^ resc^+^	[37], [44]

### Detection of ANK Genes with PCR and Southern Blot Analysis

Total genomic DNA was extracted from each *Drosophila* line according to the Holmes-Bonner method [49]. Specific primers for each of the 23 *w*Mel ANK genes were designed (Table S1), based on the sequenced *Wolbachia* strain *w*Mel and used to probe for the homologs of the 23 *w*Mel ANK genes in the 11 *Wolbachia* strains listed in Table 1. Reaction mixtures (final volume of 20 µl) contained 1x Taq reaction buffer (750 mM Tris-Cl pH 8.8, 200 mM (NH_4_)_2_SO_4_, 0.1% Tween 20), 1.5 mM MgCl_2_, 125 µM dNTPs, 10 pmol of each primer, 1 unit Taq polymerase (Promega) and 50 ng DNA. PCRs were run on a PTC-200 thermal cycler (MJ Research Inc.) with the following cycling conditions: an initial step at 94°C for 5 min, followed by 35 cycles of 94°C for 30 sec, 30 sec at a primer dependent annealing temperature, and 72°C for 3 min, plus a final extension at 72°C for 10 min.

For Southern blot analysis, 5 µg of total genomic DNA were digested with *Eco*RI. The DNA fragments were separated on a 0.8% agarose gel and blotted onto Immobilon-Ny+ filters (Millipore), according to the manufacturer’s instructions and hybridized at 68°C for 18 h in Denhardt’s solution, according to standard procedures [50]. Probes for the 23 *w*Mel ANK genes were generated by PCR in 50 µl reactions, using specific primers amplifying part of each gene (see Table S1). PCR products were purified with the QIAquick PCR purification kit (QIAGEN) and radioactively labeled with [α-^32^P] dATP (IZOTOP) using the Prime-a-gene labeling kit (Promega). A specific probe for the *Wolbachia ftsZ* cell division gene was generated (with primers ftsZ1∶5′GTATGCCGATTGCAGAGCTTG and ftsZ2∶5′GCCATGAGTATTCACTTGGCT [51]) and used as positive control.

### Sequencing of *w*Mel-like ANK Genes

The *w*Mel-like ANK genes from the different *Wolbachia* strains were PCR amplified in 50 µl PCR reactions as described above, using the primers listed in Table S1. PCR products from three independent reactions were purified with the QIAquick PCR purification kit (QIAGEN), pulled together and sequenced by Macrogen (Korea). In cases of poor sequencing quality or the presence of unpredicted multiple products, PCR products were cloned into vector pGEM-T easy (Promega). Plasmid of at least three different clones was extracted from DH5α using the Qiaprep Miniprep kit (Qiagen) and directly sequenced with primers T7 and SP6. Sequence trace files from sequencing reactions were analyzed using the DNAStar 5.0 software package.

### Analysis of Genetic Diversity

Multiple protein sequence alignments were performed and back-translated to nucleotide sequences with MUSCLE [52], as implemented in the Geneious package, version 4.0.3 [53]. Protein sequence alignment is usually preferable due to the larger protein alphabet and because it takes into account the redundancy of the amino acid codons resulting in more reliable nucleotide sequence alignment. Alignments were confirmed by visual inspection and edited manually. In order to avoid inflations, only unambiguously aligned sequences were used. Analysis of genetic diversity was performed with DNAsp, version 4.90.1 [54]. Substitution rates were estimated with Codeml, PAML 4.1 [55], using the codon substitution model of Goldman and Yang [56]. Ankyrin repeat domains were predicted by searching the sequences against the HMM profiles of the Pfam database (http://pfam.sanger.ac.uk/) and SMART (Simple Modular Architecture Research Tool; http://smart.embl-heidelberg.de/), using the default parameters [57], [58]. Exact direct repeats larger than 8 nt within ANK genes were identified with the program REPFIND [59], using a P-value cutoff of 0.0001.

### Phylogenetic Analysis

The phylogenetic relationships of the ANK genes were estimated using the maximum-likelihood (ML) method. Datasets with strong homology (>99% at nucleotide level) between the ankyrin gene orthologs were omitted from the analysis. ANK gene sequences from the A-supergroup strain *w*Uni and the B-supergroup strain *w*Pip, which infect the non-*Drosophila* hosts *Muscidifurax uniraptor* (Hymenoptera) and *Culex quinquefasciatus* (Diptera), respectively, were also included in our datasets for comparison. Prior to ML analysis, DNA substitution model parameters were estimated using Modeltest3.7 [60] and the Akaike information criterion (AIC): K81uf (WD0292, WD0766); TIM (WD0073); HKY (WD0035, WD0291, WD0441, WD0550, WD1213); TrN+I (WD0438, WD0498, WD0596); TVM+I (WD0191); TVM+G (WD0385, WD0566, WD0633, WD0754); TrN+G (WD0636); GTR (WD0147); GTR+I (WD0637). ML heuristic searches were performed using 100 random taxon addition replicates with tree bisection and reconnection (TBR) branch swapping. Bootstrap support was inferred using 100 bootstrap replicates. Searches were performed with PAUP, version 4.0b10 [61]. The ML method was also used to study the relationships between individual ankyrin repeat domains. Finally, the congruence between tree topologies was evaluated, using the SH-test [62], as implemented in PAUP 4.0b10. The SH-test compares the likelihood of score (lnL) of a given data set across its ML tree, versus the lnL of that data set across alternative topologies, which in this case are the ML phylogenies for the other ANK gene data sets [63]. The significance in differences among the likelihood scores was evaluated with a bootstrap test, using 1,000 permutations under full optimization.

### Test for Recombination

For the purpose of recombination analysis, we discarded from the ANK gene datasets all but one sequence from groups of identical sequences. Furthermore, sequences that shared less than 70% identity were also discarded in order to avoid misalignment artifacts and to minimize the probability of false positive signals. In order to increase the possibility of including potential parental or daughter sequences, ankyrin repeat gene sequences from the A-group strain *w*Uni and the B-group strain *w*Pip were also added to our datasets. To identify potential recombination events, we used the recombination detection program RDP, version 3b27 [64], which implements different methods for detecting recombination signals. We primarily used the MaxChi method [65], [66], but detected signals were considered significant only when they were confirmed by multiple methods, including Chimera [65], [66], Geneconv [67], RDP [68] and Bootscan [69]. The highest acceptable *P* value cutoff was set to 0.001, using a Bonferroni correction. Significance was evaluated with a permutation test based on 1000 permutations.

### Nucleotide Sequence Accession Numbers

All ANK gene sequences generated in this study have been deposited into GenBank under accession numbers JX839306-JX839448.

## Results

### Distribution of *w*Mel Like ANK Genes Using PCR and Southern Blot Analysis

The occurrence of the 23 *w*Mel ANK genes in the eleven different *Drosophila*-*Wolbachia* associations listed in Table 1 was investigated by PCR and Southern blot analysis (Figure S1). The results of this analysis are summarized in Table S2. The data are largely in agreement with the work of Iturbe-Ormaetxe et al. [30], which examined the distribution of the 23 *w*Mel ANK genes in nine *Wolbachia* strains [seven strains in common with the present study] (see Table S2). However, there are some differences, mostly between *Wolbachia* strains belonging to supergroup B (Table S2). Possible explanations for these variations are: (a) the different probes used in the two studies, (b) the different hybridization conditions and (c) the different primer sets used for the PCR analysis.

Only nine out of the twenty-three *w*Mel ANK genes (WD0035, WD0191, WD0438, WD0441, WD0498, WD0636, WD0637, WD0766 and WD1213) are present in all supergroup A and B *Wolbachia* strains tested, based on Southern blot analysis. However, their presence was not for all of them confirmed by PCR, probably due to variability of the primer binding sites. All twenty-three *w*Mel ANK genes were detected in the *Wolbachia* strains belonging to the *w*Mel subgroup of supergroup A (*w*MelPop, *w*Au, *w*Tei, *w*Yak, *w*San), with the exception of WD0514, which was absent from strain *w*Au. The distribution was different for the two more distantly related strains *w*Ri and *w*Ha. Both strains were tested positive for almost the same number of ANK genes (16 genes in *w*Ri and 17 genes in *w*Ha). Interestingly, the group of WO-A prophage-associated ANK genes (WD0285, WD0286, WD0291, WD0292, and WD0294) was found in *w*Ha, whereas they were absent from *w*Ri. However, the absence of these genes from *w*Ri does not imply the absence of prophage WO-A [10], [14]. Lateral phage transfer, along with the activity of transposable elements could have resulted in the differential loss or independent acquisition of ANK genes between those strains. Although we could not detect a copy of the ANK gene WD0566, the published genome sequence of strain *w*Ri [10], confirmed the presence of a highly diverged variant (∼52% pairwise identities at nucleotide level with *w*Mel). Finally, except for the nine universal genes, most ANK genes could not be detected in supergroup B strains (*w*No, *w*Mau, *w*Ma).

### Sequence Analysis Revealed ANK Gene Polymorphism

Using internal and/or external primers, we obtained partial or, in some cases, full length sequence of *w*Mel-like ANK genes from the different *Wolbachia* strains. The sequence analysis showed that some ANK genes display, beyond single nucleotide polymorphisms, variations in the number and organization of the ANK repeat domains, as well as structural disruptions, including ORF disruption by frame shift mutations and insertion of transposable elements (Table 2).

**Table 2:** Major evolutionary events shaping *Wolbachia* ANK genes.

	Ankyrin genes^a^	Variation in the ankyrin repeat architecture	Frameshift mutations	Disruption by IS	Recombination (MaxChi, *P*<0.001)
**homologs**	WD0073	Yes			Yes (*w*Uni)^b^
	WD0147	Yes			
	WD0294	Yes			
	WD0385	Yes	Yes	Yes	
	WD0438	Yes			Yes (*w*Ri)
	WD0514	Yes			
	WD0550	Yes	Yes	Yes	Yes (*w*Uni)
	**WD0566**	Yes			
	**WD0633**	Yes			Yes (*w*Au)
	**WD0636**				
	WD0754	Yes			
	WD0766	Yes	Yes	Yes	Yes
	WD1213		Yes		
**true orthologs**	WD0035				
	WD0191				
	**WD0285**				
	**WD0286**				
	**WD0291**				
	**WD0292**				
	WD0441				
	WD0498				
	**WD0596**				
	**WD0637**				

a Prophage associated ANK genes are presented in bold face.

b Strains potentially involved in recombination are shown in parenthesis.

**Figure 1:**
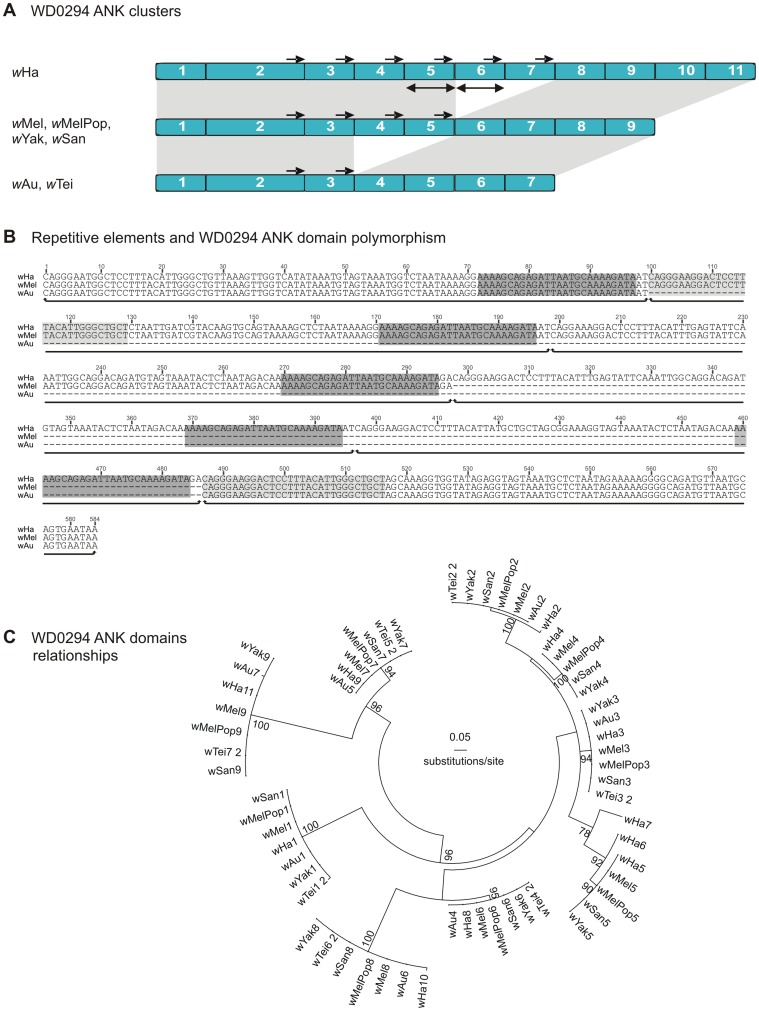
Repetitive DNA sequences and ankyrin repeat domain number polymorphism. The example of WD0294. A) Only the ankyrin repeat domain containing regions are shown. Blue rectangles represent individual ankyrin repeat domains. The light gray shading between the ANK clusters indicates homologies between the different strains. Double arrows represent identical duplications. Small black arrows indicate direct repeats capable of engaging into illegitimate recombination. B) Detail of the nucleotide alignment around the deleted region. This region includes the ANK domains 3–8 from *w*Ha, 3–6 from *w*Mel and 3–4 from *w*Au. Gray rectangles show the position of repeated sites flanging the deletions. ANK repeats are underlined. C) ML phylogenetic relationships between individual ankyrin repeat domains.

**Figure 2:**
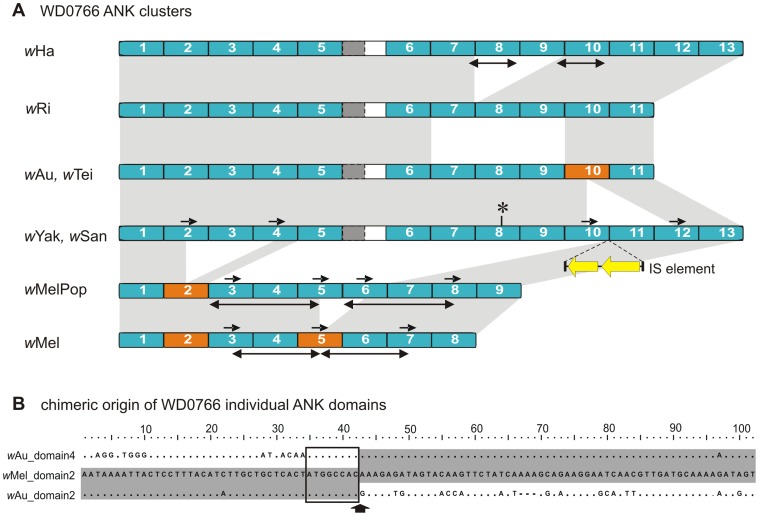
Repetitive DNA sequences and ankyrin repeat domain number polymorphism. The example of WD0766. A) Only the ankyrin repeat domain containing regions are shown. Blue rectangles represent individual ankyrin repeat domains. Dark gray rectangles with dotted outline represent ankyrin repeat domain remnants. The light gray shading between the ANK clusters indicates homologies between the different strains. Orange rectangles represent putative chimeric ankyrin repeat domains, and double arrows represent identical duplications. Small black arrows indicate direct repeats capable of engaging into illegitimate recombination. The reconstructed structure of disrupted *w*Yak and *w*San ANK homologs is also presented. The asterisk and the double yellow arrows correspond to a frame shift mutation and the position of the IS5 element, respectively. B**)** example of chimeric origin of *w*Mel (and *w*MelPop) ankyrin repeat domains 2. Identities with the parental ankyrin repeat domains 2 and 4 from *w*Au are shaded. Box shows the position of the repeated site between the three sequences. The vertical arrow indicates the loop between the two α-helices of the ankyrin repeat domain.

**Figure 3:**
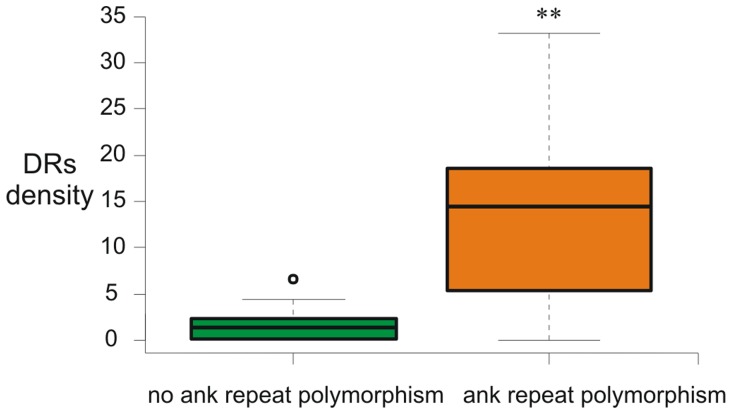
Density of direct repeats larger than 8nt per ANK gene. Box plot graph showing that ANK genes display ankyrin repeat number variations have greater DR density compared with genes that do not display variations (**P<0.005, Mann-Whitney test).

Careful visual inspection of the alignments of the *w*Mel-related ANK genes that display domain number variations revealed that short or long identical direct repeats always flanked the duplicated and/or deleted segments (Figures 1, 2 and supporting Figure S2). For example, the identified deletions in the prophage-associated WD0294-related ANK genes are flanked by two groups of perfect repetitive elements of 26 bp (AAAAGCAGAGATTAATGCAAAAGATA) and 30 bp (CAGGGAAGGACTCCTTTACATTGGGCTGCT) long (Figure 1B). This striking observation suggests that the polymorphism of the number of ankyrin repeat domains depends on the presence of repetitive sequences scattered over the ANK clusters. In order to test the hypothesis that these short repetitive sequences are implicated in the expansion and/or contraction of the ankyrin repeat domains, the density of direct repeats (number of DRs/kb) was estimated for each ANK gene. The estimation was done on direct repeats larger than 8nt with the average length being between 10–20nt (supporting Figure S3). It was also investigated whether genes with ankyrin repeat domain number variations had greater DR density compared to genes that do not display variations. The former genes were indeed found to contain significantly more direct repeats (average of 14.3 and 1.4 repeats/kb, respectively, *P*<0.005, Mann-Whitney test) (Figure 3).

Almost half of the *w*Mel-related ANK genes studied (WD0073, WD0147, WD0294, WD0385, WD0438, WD0514, WD0550, WD0566, WD0633, WD0754 and WD0766) display variations in the architecture of the ankyrin repeat domains in at least one of the studied *Wolbachia* strains (Table 2). This is particularly evident in the closely *w*Mel-related strains *w*MelPop, *w*Au, *w*Tei, *w*Yak and *w*San, in which the *w*Mel-related ANK genes share high sequence similarity at the nucleotide level; however display differences in the number of ankyrin repeat domains. The rest of the genes exhibit similar numbers and organization of ankyrin repeat domains in the different *Wolbachia* strains.

Duplications and/or deletions of ankyrin repeat domains, including shuffling between the structural elements of physically distant ankyrin repeat domains, are often the reason for copy number variations. For example, the prophage-associated WD0294-related ANK genes display high similarity at the nucleotide level in all strains which harbour it (Table S3). However, in the strains *w*Au and *w*Tei, the genes code for two ankyrin repeat domains less than those present in the strains *w*Mel, *w*MelPop, *w*Yak and *w*San. On the other hand, in the strain *w*Ha the homolog of the same gene codes for two ankyrin repeat domains more (Figure 1A). Similarly, (a) the WD0514-related ANK genes present in the strains *w*Tei, *w*Yak and *w*San code for two ankyrin repeat domains less than those found in the strains *w*Mel and *w*MelPop and (b) the WD0550-related ANK genes present in the strains *w*Au and *w*MelPop code for two ankyrin repeat domains more than those of the strains *w*Mel, *w*Tei, *w*Yak and *w*San (data not shown).

The present study confirmed that the WD0766-related ANK genes exhibit extensive variability in both number and organization of the ankyrin repeat domains (Figure 2A) as previously reported [30], [70]. In addition, sequence analysis of the WD0766-related ANK genes in the closely related *Wolbachia* strains *w*Yak and *w*San showed that these genes are disrupted due to the insertion of a full-length IS5 element (a similar phenomenon was reported for the WD0385-related ANK gene in the strain *w*Au by Iturbe-Ormaetxe et al., 2005) [30] and the presence of a frame-shift upstream of the IS element. The reconstruction of these two ORFs showed that the WD0766-related ANK genes contain 13 complete ankyrin repeat domains, two repeats longer than the WD0766-related ANK gene of the closely related strain *w*Tei and that of the *Wolbachia* strain *w*Au. It is worth noting that the 5′ and the 3′ regions of the WD0766-related ANK gene, including the first and the last ankyrin repeat domains, share high conservation between all *Wolbachia* strains studied. However, extensive duplications and deletions of the internal ankyrin repeat domains interrupt this homology.

Inspection of the multiple alignment of WD0766-related ANK gene sequences reveals that deletion events within the ANK cluster may have resulted in the shuffling of physically distant ankyrin repeat domains. One such putative case is the formation of the second ankyrin repeat domain of the *w*Mel and *w*MelPop genes. According to the alignment, this ankyrin repeat domain is chimeric. The first 42 bp, which encode the first α-helix of the ankyrin repeat domain (amino acid residues 1–14), are very similar to the corresponding region of the second ankyrin repeat domain of the WD0766-related ANK genes of strains *w*Au, *w*Tei, *w*Ri and *w*Ha, while the last 57 bp, which encode for the second α-helix (amino acid residues 15–33), are very similar to the fourth ankyrin repeat domain of the same strains (Figure 2B). Similarly, the 10^th^ ankyrin repeat domain of the *w*Au and *w*Tei WD0766-related ANK genes is probably the result of shuffling between the 10^th^ and the 12^th^ ankyrin repeat domains of the corresponding *w*Yak and *w*San genes, while the 5^th^ ankyrin repeat domain of the *w*Mel gene (WD0766) seems to be the result of a shuffling event between the 5^th^ and the 6^th^ ankyrin repeat of the *w*MelPop WD0766-related ankyrin gene (Figure 2A).

The WD0147-, WD0438-, WD0566- and WD0754-related ANK genes present in the *Wolbachia w*Mel subgroup display no ankyrin-repeat polymorphism. It is also worth noting that the partial sequences of the WD0385-related ANK genes of the *w*Mel subgroup strains, which correspond to the last two ankyrin repeat domains, have identical sequences. However, the corresponding five genes of the *w*Ri strain display extensive variations in number and/or organization of the ankyrin repeat domains, suggesting that they have undergone extensive duplications and rearrangements [10].

The prophage-associated WD0636-related ANK genes present in *Wolbachia* strains *w*Au and *w*Tei contain a premature stop codon, due to the insertion of G between position 283 and 284 of the *w*Mel sequence, eliminating the last ankyrin repeat domain of the gene together with the 3′-end of the gene (data not shown). The WD1213-related ANK genes present in the strains *w*No, *w*Mau and *w*Ma (all B supergroup strains) are identical to each other; however, they carry eleven indels, three of which cause frame shifts, compared to the WD1213 ANK gene in *w*Mel (data not shown).

Based on the above, the studied *w*Mel-related ANK genes were classified into two major groups: (a) the true orthologs, which are of the same length and have the same number of ankyrin repeat domains and (b) the homologs which differ in the length, copy number and the organization of the ankyrin repeat domains and may also carry structural disruptions, such as frame shifts, deletions and insertions.

### Genetic Diversity of ANK Genes

**Figure 4:**
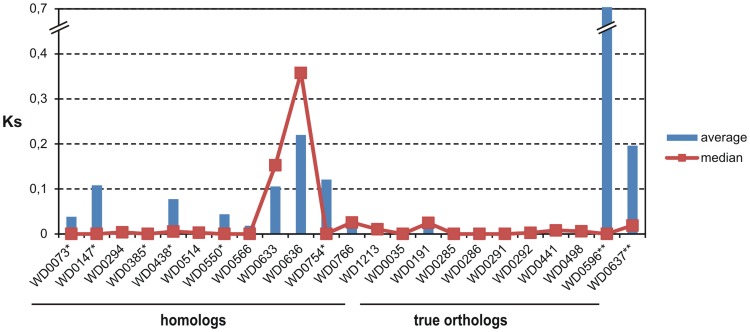
Comparative analysis of genetic diversity. The graph represents the patterns of synonymous substitutions within A-supergroup strains. (*: *w*Ri is an outlier, **: *w*Ha is an outlier).

Table S3 summarizes the features of the ANK genes detected in the studied *Wolbachia* strains, the great majority of which belong to supergroup A. The G+C content ranged from 30.8% (chromosomal ANK gene WD0438) to 42% (phage ANK gene WD0636), with an average of 35%. Overall, the genetic diversity analysis revealed high conservation at the nucleotide level across the ANK genes, as indicated by the synonymous substitutions (Ks) (Figure 4 and Table S3). In general, the majority of the ANK genes of *w*Mel-subgroup strains (*w*Mel, *w*MelPop, *w*Au, *w*Tei, *w*Yak and *w*San) are highly conserved (almost 100% identity at the nucleotide level) compared to the corresponding genes of the more distantly related strains *w*Ri and/or *w*Ha (distantly related based on MLST analysis [46], [47]), which display the highest degree of genetic variability and in some cases act as outliers.

It is worth noting that although the WD0294, WD0514, WD0550 and WD0766 homologs differ in ankyrin repeat domain architecture, they show limited sequence polymorphism (SNPs). However, the WO-B prophage-associated homologs WD0633 and WD0636 exhibit significant levels of genetic diversity (Ks>0.05) (Figure 4 and Table S3), including the two copies of the WD0636 gene detected in the *Wolbachia* strain *w*San (95% identity at nucleotide level - Ks = 0.082).

The sequences of only five (WD0441, WD0498, WD0636, WD0637 and WD01213) of the nine universally occurring ANK genes were retrieved from the three B-supergroup *Wolbachia* strains *w*No, *w*Mau and *w*Ma (Table S2) and showed almost 100% identity. The prophage-associated ANK gene WD0636 was again the exception, exhibiting a high level of genetic diversity (Ks_avg_ = 0.07535) (Table S3). Interestingly, two copies of the WD0636 gene were detected in the strain *w*No, which were 89% identical at nucleotide level (Ks = 0.15). It should also be noted that we sequenced a partial fragment (356 bp) of a highly diverged WD0191 ortholog from strain *w*Mau, which exhibited 67.2% identity to A-supergroup orthologs. Overall, there is a greater degree of genetic divergence between supergroups A and B, as indicated by the patterns of synonymous substitutions (Ks) ranging from Ks_avg_ = 0.238 (WD0636) to Ks_avg_ = 0.495 (WD0498; Table S3).

### Phylogenetic Analysis

**Figure 5:**
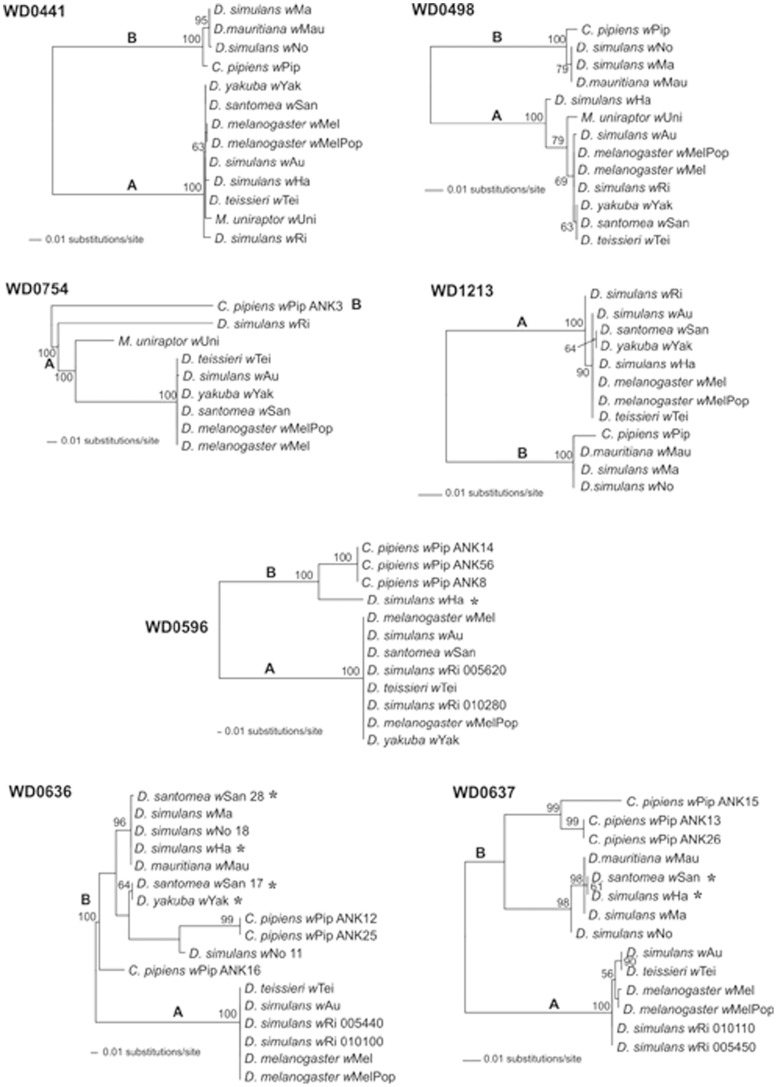
Phylogeny of four chromosomal- (WD0441, WD0498, WD0754 and WD1213) and three prophage-associated (WD0596, WD0636, WD0637) ANK genes. The trees are midpoint-rooted and inferred using maximum likelihood. ML bootstrap support values inferred from 100 replicates are also presented. Bootstrap values lower than 50 are omitted. The discordant positions of strains *w*Yak, *w*San and *w*Ha between the chromosomal- and prophage-associated ANK gene phylogenies are highlighted with asterisks. Evolutionary model parameters were estimated with Modeltest under the Akaike Information Criterion: HKY (WD0441,WD1213); TrN+I (WD0498, WD0596); TVM+G (WD0754); TrN+G (WD0636); GTR+I (WD0637).

The relationships of the ANK genes were also studied with ML phylogenetic analysis. ANK orthologs with almost 100% identity at nucleotide level (like the WO-A prophage ankyrin genes WD0285 and WD0286) were omitted from the analysis. Most trees clearly reflect the evolutionary divergence of the two major *Wolbachia* supergroups A and B; however, branch lengths separating the two supergroups are often in disagreement between the different ANK gene-based trees (Figure 5 and supporting Figure S4). The discordance suggests that the rate of evolution varies for different genes, and this is supported by the different levels of genetic diversity observed between these genes. Within supergroup A, the topologies were not significantly different between the datasets, placing all strains in a single clade. However, in some cases the more distantly related strains *w*Ri and *w*Ha branched at different positions, being separated by long genetic distances (WD0498 and WD0754 gene-based trees in Figure 5). This is supported by the highly heterogeneous pattern of genetic diversity observed in different ANK genes in different *Wolbachia* strains and could reflect different evolutionary rates or even different times of gene acquisition.

Perhaps the most interesting observation was that the two WO-B prophage-associated ANK genes WD0636 and WD0637 showed significant topological conflicts with the chromosomal ANK genes and also with other WO-B prophage-associated ankyrin genes (Figure 5). The WD0636 and WD0637 orthologs-based phylogenetic trees strongly support grouping of the two A-group strains *w*Yak and *w*San with *w*Ha and the B-supergroup strains *w*No, *w*Mau and *w*Ma. To statistically evaluate the topological incongruence, the ML phylogenies of the two prophage-associated ANK genes were compared with the ML phylogenies of the chromosomal ANK genes (WD0441, WD0498 and WD1213), using the SH test. The analysis was restricted to eight *Wolbachia* strains (*w*Mel, *w*Au, *w*Tei, *w*San, *w*Ri, *w*Ha, *w*No and *w*Mau). The likelihood-based SH test for significance of topological differences supports the discordances among topologies of the WD0636 and WD0637 gene-based phylogenies with the topologies of chromosomal ANK genes (Table 3). Interestingly, the phylogenies of WD0636 and WD0637 also showed topological incongruence (SH test *P*<0.05, data not shown) with other WO-B prophage-associated ANK genes like WD0596 and WD0633. That could be explained either by the presence of multiple prophage elements in the genomes of *w*Yak and *w*San, harbouring a different ANK gene repertoire, or due to recombination events between different prophages.

**Table 3:** Shimodaira-Hasegawa test for the statistical significance of the topological incongruence between alternative chromosomal- and prophage-associated ankyrin genes.

	Data Set				
Topology	WD0441	WD0498	WD0636	WD0637	WD1213
WD0441	**1228.38**	1054.82	497.64***	1599.62***	1196.26
WD0498	1228.56	**1046.17**	454.18***	1365.17***	1200.87
WD0636	1624.91***	1141.92***	**399.14**	1118.92	1665.61***
WD0637	1624.91***	1145.01***	409.40	**1091.34**	1653.28***
WD1213	1228.38	1054.82	497.64***	1565.64***	**1188.06**

Values are the likelihood score (-ln L) of a given data set across its own ML tree (boldface), as well as across the alternative tree topologies. Significance levels are based on full optimization.

*** *P*<0.0001.

### Recombination within ANK Genes

**Figure 6:**
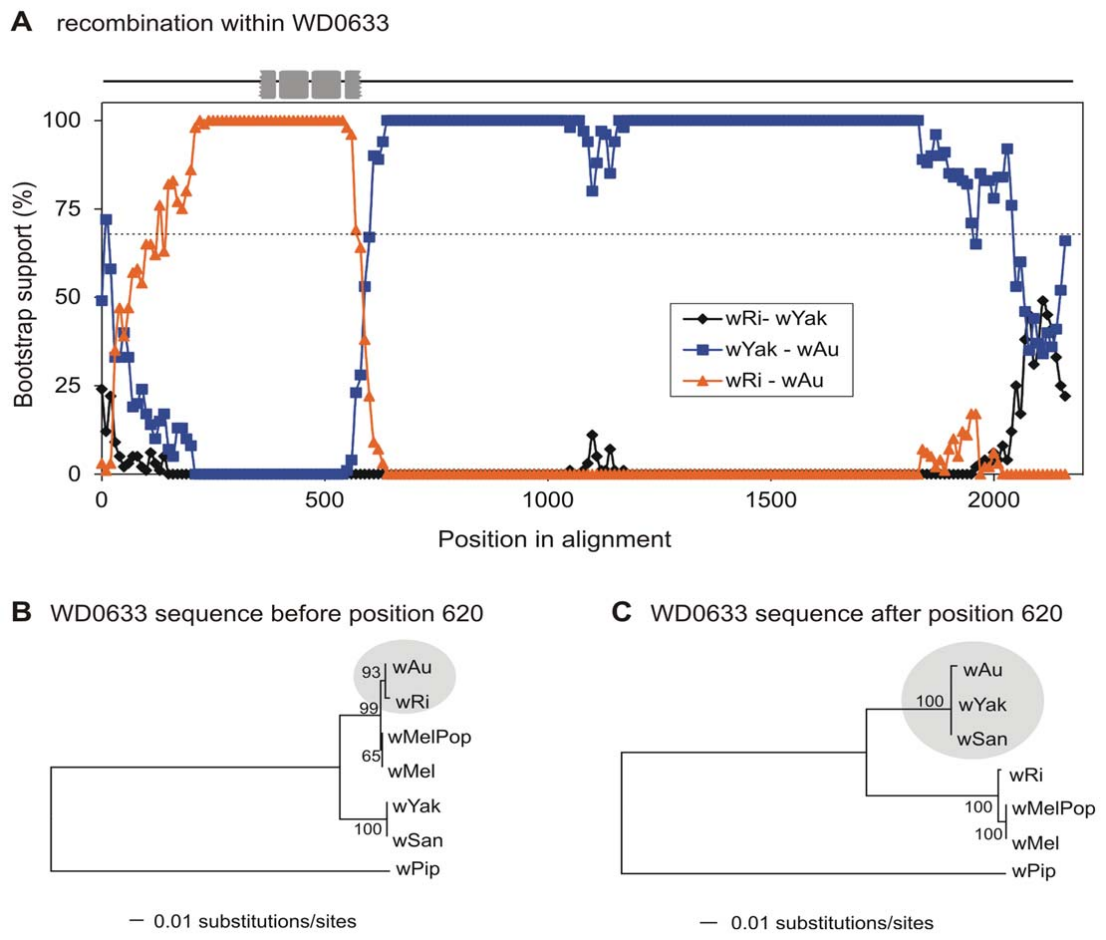
Recombination within the prophage-associated ANK gene WD0633. A) The relative bootstrap support values (1000 bootstrap resampling) are shown, calculated for a moving 200 bp window with a 10bp step size across the alignment of the WD0633 homologs. For each alignment-window, nucleotide distances and phylogenetic trees were produced using the neighbor joining method. The dotted line indicates 70% cutoff. Gray rectangles represent the positions of the ankyrin repeats in the alignment. B, C) ML phylogeny of *Wolbachia* strains reconstructed separately for the 5′ and 3′ regions of WD0633 supports the group shift of the putative recombinant strain (*w*Au).

The role of recombination in the evolution of *Wolbachia* ANK genes was investigated with the program MaxChi [65], [66]. Statistically significant recombination signals were also confirmed by the programs Chimera [65], [66], Geneconv [67], RDP [68] and Bootscan [69]. As summarized in Table 2, five ANK genes, the prophage-associated ANK gene WD0633 and the chromosomal ANK genes WD0073, WD0438, WD0550 and WD0766, exhibited significant evidence of intragenic recombination (MaxChi, *P*<0.0001 based on 1000 permutations). We present in detail only a single example, that of the prophage-associated ANK gene WD0633. A significant recombination event was detected by MaxChi, as well as by Chimera, Geneconv, RDP, and Bootscan (*P*<<0.001 based on 1000 permutations), at position 620 of the nucleotide sequence alignment. The breakpoint detected by MaxChi divides the alignment into two parts, the first of which encodes the 3^rd^ and the 4^th^ ankyrin repeat domains, demonstrating exchange of the entire ANK clusters between *Wolbachia* strains *w*Ri and *w*Au (Figure 6A). The sequence of the *w*Au homolog 5′ region with respect to the predicted breakpoint is more similar to the *w*Ri (as well as to the *w*Mel and *w*MelPop) homologs, while the sequence of the 3′ region of the breakpoint is more similar to the *w*Yak and wSan homologs. Phylogenetic trees reconstructed separately for the two regions (Figure 6B & C) clearly show the shifted position of strain *w*Au.

Our results indicate that *Wolbachia* ANK genes undergo recombination rather frequently, as it was possible to detect significant recombination signals in our small dataset. The possibility that more ANK genes have been involved in recombination events could not be excluded, as the detection of recombination signals might have been masked by extensive sequence variability and the sampling bias. This is supported by the fact that some ANK genes displayed local variations in nucleotide divergences between different strains. For example, the chromosomal ANK gene WD0441 displayed in a comparison between A- and B-supergroup strains, local conservation in the 3′ region of the gene (including the two ankyrin repeat domains) with ∼90% identity, while the 5′ region of the gene displayed extensive variability with less than 50% identity (Figure S5).

## Discussion

ANK proteins are involved in numerous and diverse processes and have been suggested to play an important role in host-symbiont interactions [17], [70]–[73]. This study investigated the presence, diversity and evolution of ANK genes in different *Drosophila*-*Wolbachia* associations. Our data show that the *Wolbachia* ANK genes form a rapidly evolving gene family and the plausible mechanisms of their evolution are classical homologous recombination, illegitimate recombination and genomic flux mediated by prophages. These data further confirm that recombination represents a powerful mechanism that accelerates and shapes genomic evolution [74]–[79].

Sequence and phylogenetic analysis of the *Wolbachia* ANK genes revealed that one of the major causes for the observed sequence polymorphism is recombination, both homologous recombination and illegitimate recombination. Intragenic recombination was observed in both chromosomal and prophage-associated ANK genes. In several cases, intragenic recombination events resulted in the exchange of entire ANK clusters.

An important finding of the present study was the positive correlation of the presence of short DRs scattered over the ankyrin repeat domains with ankyrin repeat domain number polymorphism providing an example of the mechanisms affecting ANK gene evolution. Such short repetitive sequence elements are known to play a major role in DNA deletion and duplication events in both prokaryotes and eukaryotes. Illegitimate recombination events are independent of RecA and are thought to occur by at least two different mechanisms: replication slippage and single-strand annealing [80], [81]. The presence of DRs in the same location with respect to ankyrin repeat domains is important in order to maintain reading frame, structure and hence the function of the ankyrin repeats. Thus, the direct repeats involved in illegitimate recombination, duplication, deletion as well as recombination between distant ankyrin domains, likely play an important role in evolution of *Wolbachia* ANK genes. Recombination events mediated by short repeated sequences scattered over genes may have resulted in gene deterioration and the production of species-specific orphans, including a putative ANK gene, in the closely related *Rickettsia* species, *R. conori*, *R. rickettsii* and *R. montana* [82].

It is also important to note that stress-response genes in prokaryotes are known to have a higher than average number of short or large repeats capable of engaging in recombination, probably as a strategy to cope with unstable environmental conditions [83]. One could speculate that the ANK genes may have a similar role in *Wolbachia* or they may determine the host range and/or tissue tropism, like the ANK proteins of eukaryotic viruses [84]. In addition, it has been suggested that the evolutionary success of the eukaryotic ANK protein family is in part due to their ability to bind to multiple targets by adapting their binding sites through duplication, deletion and shuffling, as a result of alternative exon splicing [71], [72], [85], [86]. It is also believed that modular proteins, like the ANK proteins, evolve faster than non-modular proteins through recombination [72], [86]. Illegitimate recombination, as well as homologous recombination, can act within one genome (including the ANK genes) providing a source of genetic variability needed for *Wolbachia* strains to rapidly adapt to new environmental conditions.

### Bacteriophage Flux and Evolution of *Wolbachia* ANK Genes

Bacteriophages are major determinants in bacterial genome evolution [87]–[89]. *Wolbachia* prophage elements are abundant and widespread and can laterally transfer between different *Wolbachia* strains co-inhabiting the same arthropod host [15], [63], [90]–[93]. A recent study reported that the prophage-associated ANK genes form one of the most divergent groups of *Wolbachia* genes [14]. Our analysis supports this finding and indicates that the ANK gene “cargo” of a given prophage may differ between strains, as is the case for the five WO-A associated ANK genes that seem to be an independent acquisition in *w*Ha and the *w*Mel-like strains. Furthermore, there is an absence of congruence between the phylogenies of prophage WO-B associated ANK genes not only with the chromosomal but also with other WO-B associated ANK genes. This incongruence is indicative of an active phage, which is able to move horizontally between different *Wolbachia* strains sharing a common host. This mechanism of genetic exchange was previously suggested for *Wolbachia* strains including *w*Ha and is known as the “intracellular arena” hypothesis [63], [91]. Lateral phage transfer coupled to intra- and intergenic recombination events could account for this rapid exchange and spreading of ANK genes.

### Multiple Phages vs Multiple Infections

Our analysis of the 23 *w*Mel-like ANK genes in different *Drosophila*-*Wolbachia* associations revealed striking differences between the strains, as well as between the genes studied. This was particularly evident for the prophage-associated ANK genes, suggesting that the two ANK groups (chromosomal and phage) have different evolutionary histories. However, conclusions about the evolutionary history of some genes may not be easily drawn. For example, the *Wolbachia* strains *w*San (supergroup A) and *w*No (supergroup B) contain two copies of the prophage-associated ANK gene WD0636. It is not clear if the two copies are the products of a gene duplication event or belong to different copies of the WO-B prophage. The existence of multiple WO-B prophages has already been described in strain *w*Ri, which harbors two identical copies of a WO-B-like prophage [10], as well as in strain *w*Pip, which harbors five WO-B-like prophage regions [9].

It was recently shown that another prophage-associated gene, the DNA adenine methylatrasnferase gene *met2*, is present in two copies in the symbiotic association between *D. teissieri* and *w*Tei; *w*Tei being a strain closely related to *w*San. Molecular analysis indicated that the two *met2* gene copies are present in two different *Wolbachia* strains which co-exist in the host *D. teissieri* [94]. Coinfection may thus explain multiple copies of prophage-associated ANK gene WD0636 in the hosts *D. santomea* and in *D. simulans* Noumea. Although the presence of a double infection is rather a speculation for *D. santomea* (but see also below), it may indeed be the case for *D. simulans* Noumea. This host was originally doubly infected with two *Wolbachia* strains, *w*No and *w*Ha, before it was established as a *w*No mono-infected line through selection [95]. However, recent sequencing of the *Wolbachia* strain(s) infecting *D. simulans* Noumea also suggested the presence of *w*Ha in low quantities (supergroup A) [Ellegaard, Klasson, Näslund, Bourtzis and Andersson, unpublished data]. A PCR analysis may thus report genes present in *w*Ha but not in *w*No as present in *D. simulans* Noumea because of the slight contamination with *w*Ha. Indeed, the identification of WD0285 in *w*No (Table S2) is an artifact of the presence of this gene in *w*Ha, which is co-infecting at a low concentration.

Riegler et al. [70] recently proposed that polymorphic variable number tandem repeats and ANK genes can be used as a new diagnostic tool for genotyping *Wolbachia* strains. They also suggested two variable ANK genes (WD0766 and WD0550) for fingerprinting and discrimination between closely related *Wolbachia* strains belonging to supergroup A, including strains analyzed in the present study. Although the results of the two studies are largely in agreement, two differences deserve clarification. According to Riegler and colleagues, the WD0766-like ANK gene in the *w*Tei strain is disrupted by an IS5 element, as is also the case for strains *w*Yak and *w*San. This observation is based on PCR amplicon size similarities between the three stains. Only the *w*San gene copy was sequenced. Our sequence analysis clearly demonstrates that the WD0766-like ANK gene of *w*Tei is identical to that of *w*Au. Furthermore, the number of ankyrin repeat domains of the WD0550-like ANK gene of *w*San differs between the two studies (eight ankyrin repeats in Riegler et al.*vs* six in the present study). While the first difference in WD0766 between the two studies was confirmed, it was raised that for WD0550 *w*San was erroneously listed with 8 ankyrin repeat domains in Riegler et al. [70] when it only has 6 (personal communication M Riegler, I Iturbe-Ormaetxe, WJ Miller). There are two possible explanations for the discrepancy regarding WD0776. First, the presence of hidden multiple infections with different infection levels in the *Drosophila* stocks could account for these differences. As discussed above, there is evidence supporting the presence of more than one *Wolbachia* strain in *D. santomea*, as recently documented for the closely related species *D. teissieri* [94]. An alternative explanation could be different evolutionary events in the two stocks, which could account for the observed differences. Also, *Wolbachia* IS elements are quite active, and frequent IS5 polymorphisms have been documented for *w*Mel strains [96], *w*Pip strains [97] and across a range of different A-group *Wolbachia* strains [30], which could also play a role [30], [96]–[98]. These hypotheses can be tested by further analyzing the WD0766-like ankyrin gene in *w*Tei and related strains used in both studies.

### *Wolbachia* Ankyrin Genes: Unknown Origin and Function

Earlier studies also highlighted the genetic diversity between *Wolbachia* ANK genes, but restricted themselves, however, to an attempt to correlate the observed genetic variability with different CI patterns [30], [32]. According to our results, a correlation between *Wolbachia* phenotypes and distribution or genetic polymorphism of the different ANK genes is not obvious. Papafotiou et al. [31] showed that two ANK genes, WD0438 and WD1213, show higher expression levels in testes than in ovaries; however, the authors did not detect any evidence that this sex-specific expression is related to CI.

The origin of the *Wolbachia* ANK genes remains unclear. It has been suggested that prokaryotic ANK genes were acquired from a eukaryotic host rather than evolved independently [99]. However, the discovery of ANK genes in archaea and free-living bacterial species suggests a more ancient origin [19]. The largest numbers of ANK genes in prokaryotes were found in the genomes of *Coxiella burnetii, Legionella pneumophila, Rickettsia bellii, Rickettsia felis, Orientia tsutsugamushi* and sponge symbiotic bacteria residing within eukaryotic host cells [100]–[106].The presence of a large number of ANK proteins within the genomes of these bacteria may be related to their unique lifestyle. This may also be the case for *Wolbachia*. Despite the fact that several studies investigated the direct or indirect association of the ANK genes with *Wolbachia-*induced reproductive phenotypes, mainly with cytoplasmic incompatibility, a functional correlation remains elusive [29]–[32], [70]. The present study does not shed more light on this either. However, our results strongly indicate that phage transfer, homologous recombination and illegitimate recombination have provided *Wolbachia* with a unique repertoire of ANK genes. Their role for the lifestyle of *Wolbachia* remains to be established.

## Supporting Information

Figure S1 **Example of Southern blot analysis.** Each membrane was hybridized simultaneously with two probes: a probe specific for the ANK gene under study and a probe specific for the *fts*Z gene, which was used as a positive control. A) WD0441 and B) WD0294. 1: *w*Mel, 2: *w*MelPop, 3: *w*Au, 4: *w*Tei, 5: *w*Yak, 6: *w*San, 7: *w*Ri, 8: *w*Ha, 9: *w*No, 10: *w*Ma, 11: *w*Mau.
(TIFF)

Figure S2 **Repetitive DNA sequences.** Alignments of partial fragments of ANK genes present ankyrin repeat domain polymorphism (A) WD0385, (B) WD0514 and (C) WD0550. Gray rectangles show the position of repeated sites flanging the deletions. ANK repeats are underlined.
(TIF)

Figure S3 **Abundance of DRs within ANK genes.**
(TIF)

Figure S4 **Phylogeny of the ANK genes.** The trees are midpoint rooted and inferred using maximum likelihood. ML bootstrap support values inferred from 100 replicates are also presented. Bootstrap values lower than 50 are omitted. Evolutionary model parameters were estimated with Modeltest under the Akaike Information Criterion: K81uf (WD0292, WD0766); TIM (WD0073); HKY (WD0035, WD0291, WD0550); TrN+I (WD0438); TVM+I (WD0191); TVM+G (WD0385, WD0566, WD0633); GTR (WD0147).
(TIF)

Figure S5 **Local variation in nucleotide divergences within WD0441-like ANK gene sequences.** A sliding window analysis of genetic distance between A- and B- supergroup strains indicates that the two supergroups share more similarities in the 3′ end, which includes the ankyrin repeat domains.
(TIFF)

Table S1 **Primers used in this study.**
(DOCX)

Table S2 **Distribution of** ***w*** **Mel-like ANK genes in different** ***Drosophila-Wolbachia*** **associations.**
(DOCX)

Table S3 **Comparison of** ***Wolbachia*** **ankyrin repeat genes.**
(XLSX)
