# Supplementary figures and images for: The Diversity and Evolution of Wolbachia Ankyrin Repeat Domain Genes
Source: PLoS One. 2013 Feb 4;8(2):e55390. doi: 10.1371/journal.pone.0055390 (PMC3563639; doi:10.1371/journal.pone.0055390)

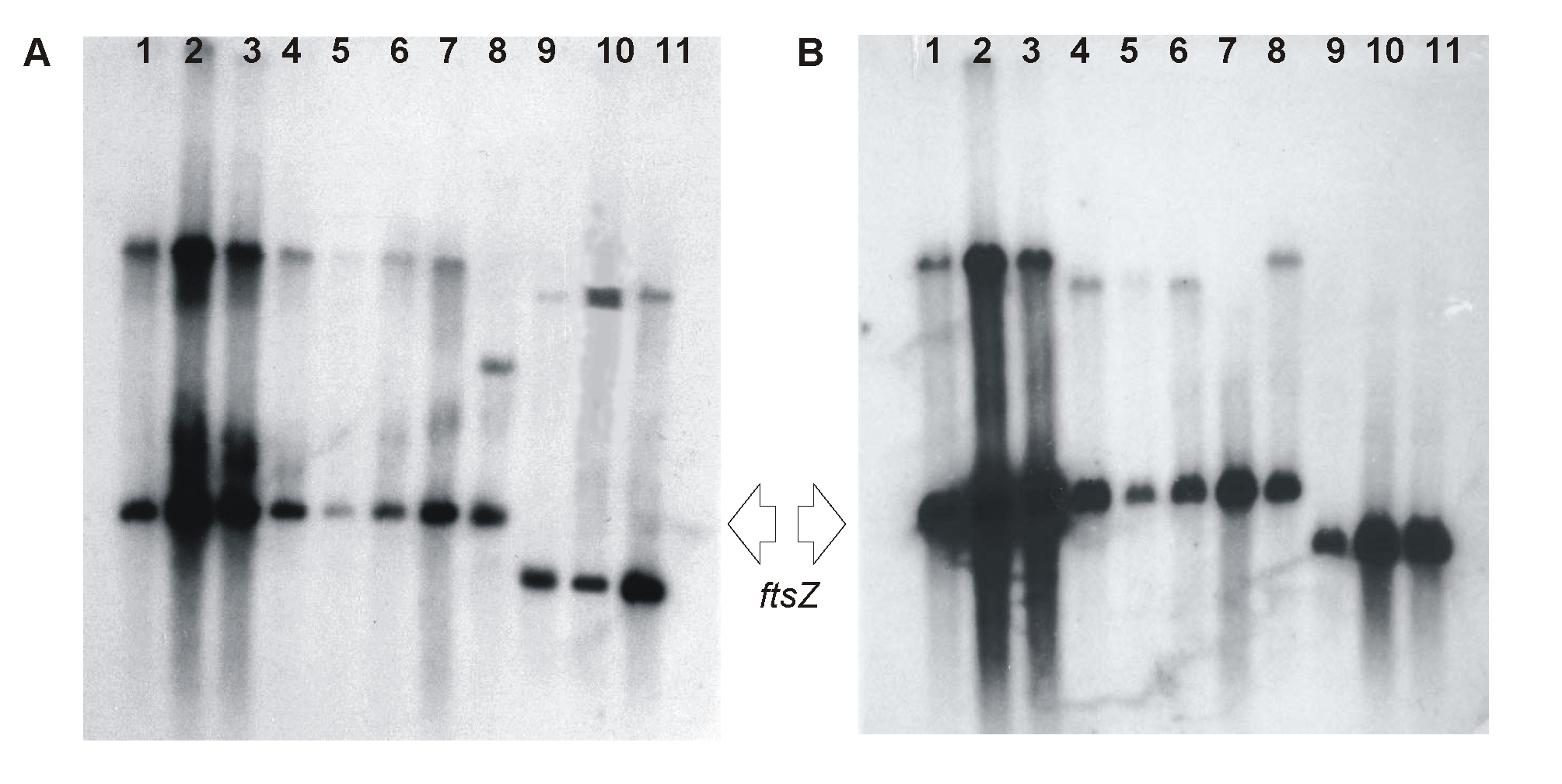

Supplement: Figure S1 — Example of Southern blot analysis. Each membrane was hybridized simultaneously with two probes: a probe specific for the ANK gene under study and a probe specific for the ftsZ gene, which was used as a positive control. A) WD0441 and B) WD0294. 1: wMel, 2: wMelPop, 3: wAu, 4: wTei, 5: wYak, 6: wSan, 7: wRi, 8: wHa, 9: wNo, 10: wMa, 11: wMau. (TIFF) [file pone.0055390.s001.tiff]

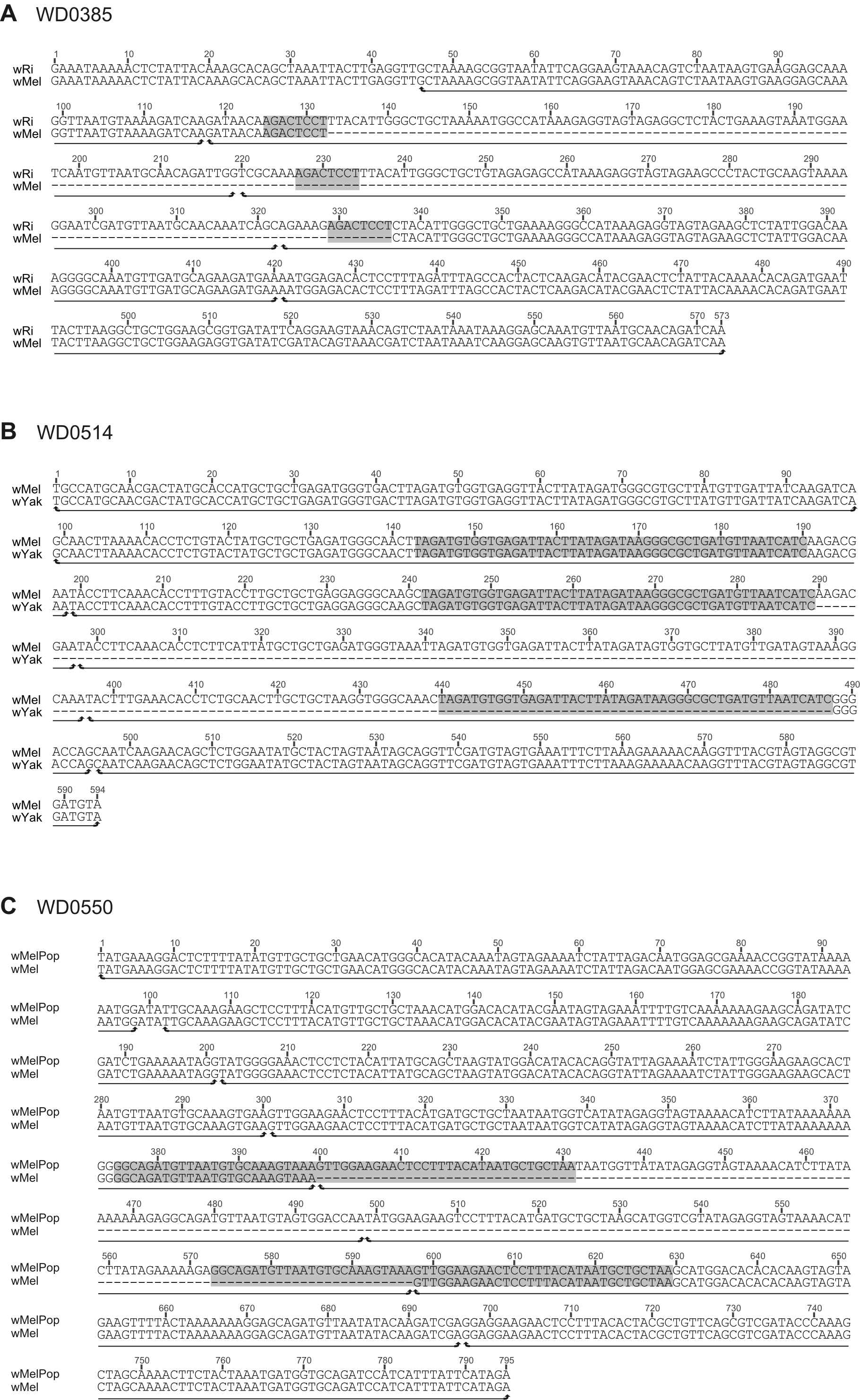

Supplement: Figure S2 — Repetitive DNA sequences. Alignments of partial fragments of ANK genes present ankyrin repeat domain polymorphism (A) WD0385, (B) WD0514 and (C) WD0550. Gray rectangles show the position of repeated sites flanging the deletions. ANK repeats are underlined. (TIF) [file pone.0055390.s002.tif]

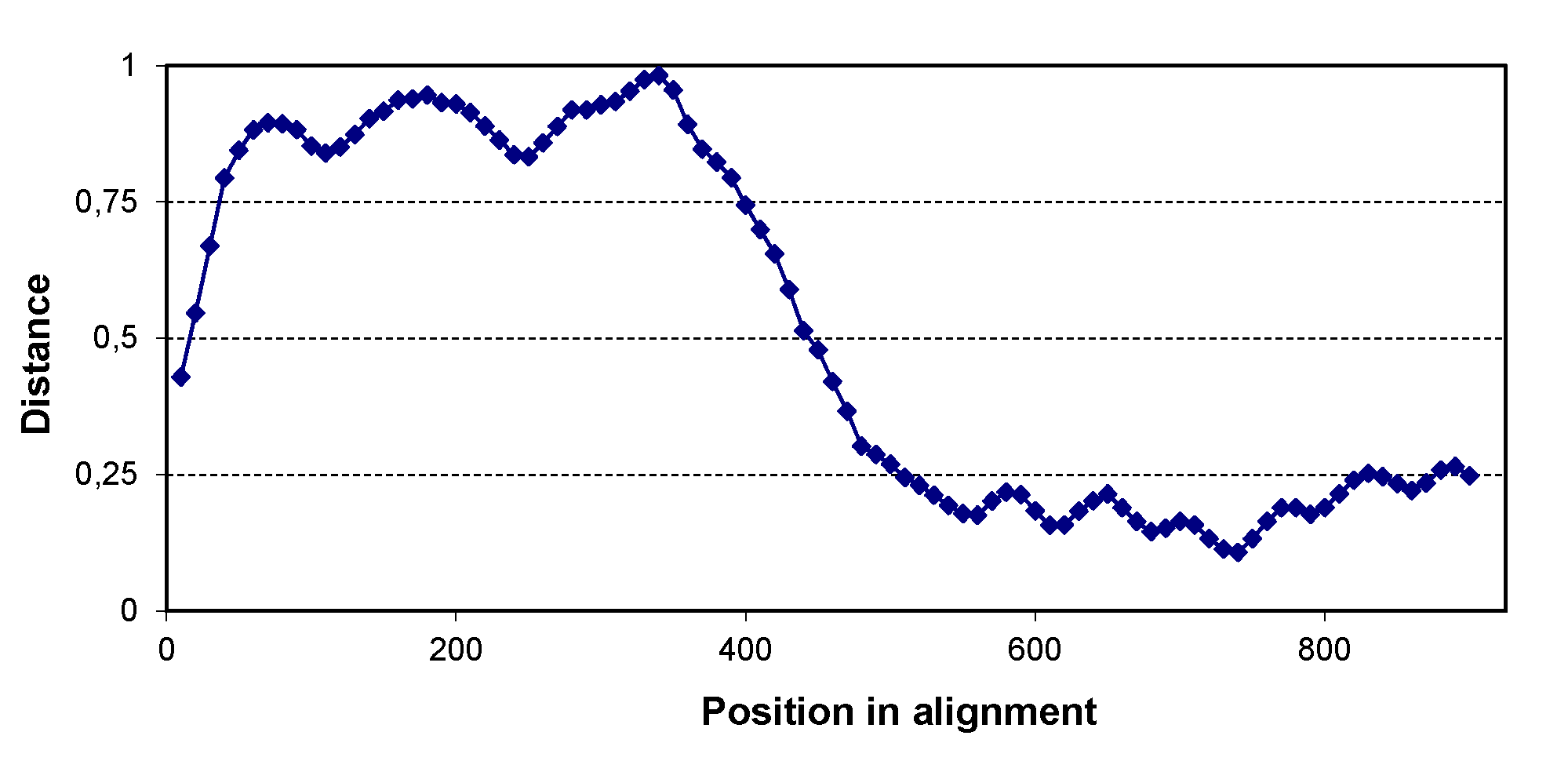

Supplement: Figure S5 — Local variation in nucleotide divergences within WD0441-like ANK gene sequences. A sliding window analysis of genetic distance between A- and B- supergroup strains indicates that the two supergroups share more similarities in the 3′ end, which includes the ankyrin repeat domains. (TIFF) [file pone.0055390.s005.tiff]
