# Supplementary material for: The Diversity and Evolution of Wolbachia Ankyrin Repeat Domain Genes
Source: PLoS One. 2013 Feb 4;8(2):e55390. doi: 10.1371/journal.pone.0055390 (PMC3563639; doi:10.1371/journal.pone.0055390)
Supplement: Table S1 — Primers used in this study. (DOCX) [file pone.0055390.s006.docx]

**Table S1.** Primers used in this study.

| **Ankyrin gene** | **primer F** | **primer R** |
| --- | --- | --- |
| WD0035 | CCATCGGACGCAAACAAT | AGCGCAATATCCAAAGCATC |
| WD0073 | GGTGCAAAACATCAGGAAGAG | CTGCAATAGCCCAATGTAAATG |
| WD0147 | TTGTGGCTCATATTATTTCTCAT | CTTTCTTTACCCCTCGTTCTAT |
| WD0191 | AGAGGGAAATACATTACTACATCA * | ATCGCTTTAGCCTGTTCATAG * |
|  | ATTTTCCCGTCGATAATTCA | TGGTGTGTGTGTTGCAGTAA |
|  | AAATACATGGCCAGTCCAAT | AGGAAAGAGAGATCGGGTTT |
| WD0285 | TGCAGGCAAATTGTCTAAAAA | ATACCTCCCTTGTCTCTTCATCAC |
| WD0286 | TTAGTAAGAAAGAAAGAGAAG | TTTTGCTACTATCACACC |
| WD0291 | CCGATACATCTGTCACTTTTG | AAGCAAGAGATCAAGGGATG |
| WD0292 | ATCGCGCTTCTGCTATGG | TTGGCTGTAAGGTCTGTGTAATG |
| WD0294 | ATGGCCATGAAGAGATAGTA * | ATTAGCGGCTGATGATTG * |
|  | ACCGTCACTATTTCTTGCGT | TAATATTGGTGGCCAAGGTC |
| WD0385 | TACTGGGAGCAAAAGAAATCAATG | CCCCAGGTGAAGAAGAATCATC |
| WD0438 | GCGATGCTGGTGATGATTATG | GCGCCAGAGAAGAGGGTTTAT |
| WD0441 | TCCAAGGACCGCAATAGAGTAA * | TCAGCACCTTGTAATAAGAGAAAG * |
|  | TATCGAAAAGCAACACTGATG | CAACATCATTAACAGTAGAACT |
| WD0498 | ATGGCGCGTGAGGAAGA | CGCAATCTAACGCATCAAAAC |
| WD0514 | ATACAGTGGCCGGATAAATAAC * | AGCACCAACAACTAAACTTCTCA * |
|  | TTCCTAACACGTCAGCAAAA | TTATGCCATGCAACGACTAT |
|  | GTTTTAAGTTGCTGATCTTG | CGGTGAGCTGGTAGAAGTTT |
| WD0550 | TACAACGGGTGGAAAGATAACAAG | ATACGCAGCCCCATAGGAA |
| WD0566 | TATAATGAAAGAGATAAACTACA | CAGCATTATAGAGCAAGTG |
| WD0596 | CGTTACACTTAGCGGCAGGAG * | AACCCCAGCATTAGGAACATTTAT * |
|  | CGGCAATAAGAGATCCAAGT | AACATAAGTCCCTTTCGTCG |
|  | TTAGAGAAGGAAATGCGGAC | CTGCACTCACTTGTTTTCTCA |
| WD0633 | CTCGGTGGCTACTCGGTCTACAA * | CCACGATTTACGAGCTCACTTTCA * |
|  | AGAATTGCAAAGGCTGCTAC | ACTCTGGAGGATTGTCTTGC |
|  | CCTGATGACTTCTATCTAGGGC | ACAGAATCAATTATGCGAAAA |
| WD0636 | AGAAAAAGAGGATGAACTAAAAG | GCATAATCCATCGCTGTCATC |
| WD0637 | TGGGAAAGTGAAAAGTATTATCTA * | ATTTCGTTTACTTGGCACATTATC * |
|  | AAGATGGGCCAGTTTTATCA |  |
| WD0754 | ATGGAGGGGATGGGATAAATACTA | GTCGCTTGCTCTTCCATACACA |
| WD0766 | AAGAGTAAACCTGACAACGATAG | TGGTACAGATAGGGCAGTAG |
| WD1213 | ACCAGCAGAGATAATTCTTCACC | TTTCTGCCTTATTTCTTCTACACT |

Asterisks indicate the primers used for probe generation.
