# Supplementary material for: The Diversity and Evolution of Wolbachia Ankyrin Repeat Domain Genes
Source: PLoS One. 2013 Feb 4;8(2):e55390. doi: 10.1371/journal.pone.0055390 (PMC3563639; doi:10.1371/journal.pone.0055390)
Supplement: Table S2 — Distribution of w Mel-like ANK genes in different Drosophila-Wolbachia associations. (DOCX) [file pone.0055390.s007.docx]

**Table S2**. Distribution of *w*Mel-like ANK genes in different *Drosophila-Wolbachia* associations.

|  | **Supergroup A** | | | | | | | | **Supergroup B** | | |
| --- | --- | --- | --- | --- | --- | --- | --- | --- | --- | --- | --- |
| Ankyrin genes | *w*Mel^a^ | *w*MelPop^a^ | *w*Au^a^ | *w*Tei | *w*Yak | *w*San | wRi^a^ | *w*Ha^a^ | *w*No^a^ | *w*Mau^a^ | *w*Ma |
| WD0035^b^ | P | P | P | P | P | P | P | P | **S** | **S** | S |
| WD0073 | P | P | P | P | P | P | P | S | A | A | A |
| WD0147 | P | P | P | P | P | P | P | A | A | A | A |
| WD0191^b^ | P | P | P | P | P | P | P | P | **P** | **P** | P |
| WD0285 | P | P | P | P | P | P | A | P | **S** | A | A |
| WD0286 | P | P | P | P | P | P | A | P | **A** | A | A |
| WD0291 | P | P | P | P | P | P | A | P | A | A | A |
| WD0292 | P | P | P | P | P | P | A | P | A | A | A |
| WD0294 | P | P | P | P | P | P | A | P | A | A | A |
| WD0385 | P | P | P | P | P | P | P | **A** | **S** | A | A |
| WD0438^b^ | P | P | P | P | P | P | P | S | **S** | **S** | S |
| WD0441^b^ | P | P | P | P | P | P | P | P | **P** | **P** | P |
| WD0498^b^ | P | P | P | P | P | P | P | P | **P** | **P** | P |
| WD0514 | P | P | A | P | P | P | A | A | A | A | A |
| WD0550 | P | P | P | P | P | P | P | A | A | A | A |
| WD0566 | P | P | P | P | P | P | A^c^ | A | A | A | A |
| WD0596 | P | P | P | P | P | P | P | **P** | **A** | A | A |
| WD0633 | P | P | P | P | P | P | P | **S** | A | A | A |
| WD0636^b^ | P | P | P | P | P | P | P | P | P | P | P |
| WD0637^b^ | P | P | P | P | P | P | P | P | P | P | P |
| WD0754 | P | P | P | P | P | P | P | A | A | A | A |
| WD0766^b^ | P | P | P | P | P | P | P | P | **S** | **S** | S |
| WD1213^b^ | P | P | P | P | P | P | P | P | **P** | **P** | P |

P Presence of an ANK gene, as determined by both PCR and Southern blot analysis

A Absence of an ANK gene, as determined by both PCR and Southern blot analysis

S Presence determined only by Southern blot analysis, but not confirmed by PCR

^a^ Strains also analyzed by Iturbe-Ormaetxe et al. [30].

Disagreements between the present and the study of Iturbe-Ormaetxe et al. [30] are presented in bold phase and underlined.

^b^ Ubiquitous ANK genes.

^c^ The recently published genome sequence of strain *w*Ri [10] confirmed the presence of a highly diverged variant.
